# Intention to Leave Among Quality Nurses in Public Hospitals in Kuwait: A Sequential Explanatory Mixed-Methods Study

**DOI:** 10.3390/healthcare14172669

**Published:** 2026-08-22

**Authors:** Talal ALFadhalah, Shaimaa Ali, Marjan Lari, Hamad Al Kharji, Hossam Elamir

**Affiliations:** 1 Quality and Accreditation Directorate, Ministry of Health, Kuwait City 13001, Kuwait; talfadalah@gmail.com; 2 Nursing Department, Quality and Accreditation Directorate, Ministry of Health, Kuwait City 13001, Kuwait; janasaad99@yahoo.com (S.A.); mlari@moh.gov.kw (M.L.); 3 Research and Technical Support Department, Quality and Accreditation Directorate, Ministry of Health, Kuwait City 13001, Kuwait; hmdkharji@gmail.com

**Keywords:** job satisfaction, nurse turnover, occupational stress, personnel retention, TIS-6, work environment

## Abstract

**Highlights:**

**What are the main findings?**

- This first nationwide mixed-methods study in Kuwait found a high level of turnover intention among quality nurses working in public hospitals, with work-related factors emerging as the most commonly reported reasons for intending to leave.
- Qualitative findings indicated that organizational and workplace conditions—including lack of organizational support, poor communication, role ambiguity, workload, emotional exhaustion, and perceived inequity in compensation and benefits—were perceived by participants as important contributors to turnover intention, whereas predictable duty hours emerged as a potential factor supporting participants’ willingness to remain in their current roles.

**What are the implications of the main findings?**

- Healthcare organizations should prioritize system-level retention strategies that improve workplace culture, leadership support, workload management, communication, professional development, and compensation for quality nurses.
- Addressing reported workplace concerns and maintaining predictable duty arrangements, where feasible, might support retention and the sustainability of quality management, accreditation, and patient safety initiatives in public hospitals.

**Abstract:**

**Background/Objectives**: In Kuwait, a marked decline in the number of quality nurses has been observed; however, the factors underpinning their intention to leave have never been investigated. This study aimed to determine the prevalence of turnover intention among quality nurses in Kuwait, describe the reported reasons for leaving, and explore the organizational and personal experiences underlying their intentions. **Methods**: A sequential explanatory mixed-methods design was used. The quantitative phase involved a total population survey of all eligible quality nurses working in public hospitals (*n* = 65), using a structured questionnaire that included demographic characteristics, the Turnover Intention Scale (TIS-6), and literature-informed reasons for leaving. Descriptive analyses and non-parametric group comparisons (Mann–Whitney U and Kruskal–Wallis H tests) were conducted. The qualitative phase comprised in-depth semi-structured interviews with seven nurses who voluntarily reported an intention to leave and agreed to explain and contextualize the quantitative findings. Interview data were analysed using inductive thematic analysis informed by phenomenological principles. **Results**: The mean turnover-intention score was 21.7 (SD = 3.9), exceeding the TIS-6 scale midpoint of 18. Overall, 54 of 65 participants (83.1%; 95% CI: 72.2–90.3%) scored above the TIS-6 midpoint of 18, consistent with the original developer’s scoring guidance for a desire to leave. The most frequently reported reasons for leaving were low salary (60.0%), stressful or unsupportive work environments (49.2%), and lack of recognition (44.6%). The qualitative findings expanded these results by illustrating how organizational factors were manifested in practice through poor communication, lack of managerial support, role ambiguity, blame culture, verbal mistreatment, emotional exhaustion, and perceived inequity. Three overarching themes were identified: organizational and workplace factors, compensation and benefits, and personal reasons. Predictable morning duty also emerged as a factor that participants perceived as supporting their willingness to remain in their roles. **Conclusions**: Turnover intention among quality nurses in Kuwait was reported primarily in relation to organizational and work-related factors rather than demographic characteristics. The qualitative findings contextualized the high turnover-intention scores by illustrating participants’ workplace experiences. These findings suggest that strengthening organizational support, clarifying professional roles, promoting equitable management practices, and reviewing remuneration structures might contribute to improving retention among quality nurses.

## 1. Introduction

The healthcare industry relies heavily on the competence and expertise of its workforce. Accordingly, attracting and retaining skilled professionals is essential for a healthcare organization [1]. To improve quality and safety, maintain regulatory compliance, manage performance data, foster a culture of harm prevention, and support continuous improvement, healthcare organizations depend on a cadre of highly skilled quality professionals [2].

Quality officers are specialized professionals who embody an organization’s commitment to quality and excellence [3]. In Kuwait’s public hospitals, quality nurses are registered nurses assigned not to routine bedside care, but to quality-management roles, including quality improvement, accreditation, regulatory compliance, performance monitoring, patient safety, and clinical audit activities [4]. As this is not a clinical nursing specialty, only relatively few nurses are assigned full-time to these responsibilities within a hospital. Although quality nurses are operationally based in public hospitals, they are functionally affiliated with the Quality and Accreditation Directorate, which oversees reporting and routine assignment to standard work tasks.

A workforce shortage occurs when there are insufficient qualified individuals in the right place at the right time to perform the required tasks [5]. Healthcare workforce shortages are a global concern, with projections indicating a deficit of 10 million healthcare professionals by 2030 [6]. Such shortages compromise access to health services and the quality of patient care [5].

High turnover among skilled healthcare professionals has important economic and organizational consequences [7]. These include increased workloads for remaining staff, reduced employee well-being, disruptions to service continuity, and substantial direct and indirect costs associated with recruiting and training replacements. The financial cost of replacing a nurse has been estimated to reach up to three times the employee’s annual salary [7]. Ultimately, high turnover in the healthcare sector can compromise the consistent delivery of safe, high-quality patient care [8].

Turnover intention refers to an employee’s conscious intention or willingness to leave their current position within a specified period [9,10]. Although it does not necessarily result in immediate resignations, it is a strong predictor of an organization’s actual turnover [9]. Consequently, examining turnover intention is an important step in understanding and preventing workforce attrition [10,11].

Previous research has consistently shown that nurses’ turnover intention is influenced by multiple interacting factors [12,13]. Organizational factors, including workload, work environment, managerial support, professional recognition, remuneration, and career development opportunities, are among the most consistently reported determinants. Individual and professional characteristics, such as work–life balance, role expectations, and years of experience, have also been associated with turnover intention, although findings have been less consistent across settings. Those observations were made in surveys of nurses regardless of type; however, the case for surveying quality nurses separately is strong as their role is so different from that of a bedside nurse. The recent post-pandemic literature continues to identify safe staffing, supportive work environments, effective leadership and communication, psychological well-being, professional development opportunities, and equitable compensation as key influences on healthcare workforce retention [14,15,16].

Although previous research has examined turnover intention among nurses in Kuwait [17], no published study has specifically investigated turnover intention among quality nurses. International studies have focused primarily on bedside and clinical nurses [18,19], including those conducted in Gulf countries [20], whereas evidence relating specifically to nurses working in quality-management roles remains limited. This represents an important knowledge gap because quality nurses play a central role in quality improvement, accreditation, regulatory compliance, and patient safety initiatives. In Kuwait, shortages of healthcare professionals continue to challenge the public healthcare system, and the increasing turnover of quality nurses has become a growing concern for nursing administration. According to Ministry of Health statistics, the turnover rate among quality nurses increased markedly from 1.1% in 2017 to 20.9% in 2022 [21].

Against this backdrop, this study aimed to determine the prevalence of turnover intention, identify the reported reasons for the intentions to leave, and explore the experiences underlying these intentions among quality nurses working in public hospitals in Kuwait. Specifically, the study addressed two research questions: (1) what proportion of eligible quality nurses had six-item Turnover Intention Scale (TIS-6) scores indicating an intention to leave? and (2) what organizational, work-related, compensation-related, and personal experiences help explain their intention to leave? By focusing on this little-studied professional group, the present study extends the existing literature and provides evidence to inform workforce planning and retention strategies in Kuwait’s public healthcare system.

To analyze our findings, the Job Demands–Resources (JD–R) model [22] was adopted as the conceptual framework for interpreting the study findings. Within this framework, workload, role ambiguity, and emotional exhaustion can be viewed as job demands, whereas managerial support, professional recognition, career development opportunities, and predictable duty hours represent job resources that might influence turnover intention.

## 2. Materials and Methods

A sequential explanatory mixed-methods design was adopted, comprising quantitative and qualitative components. The quantitative phase measured the prevalence of nurses’ intention to leave and described their reported reasons for leaving, whereas the qualitative phase explored participants’ experiences to explain and contextualize the quantitative findings. Integration of the two occurred during development of the qualitative interview guide, which was informed by the quantitative findings, and during interpretation, for which the qualitative findings were used to explain and contextualize the quantitative results. The study is reported in accordance with the Good Reporting of a Mixed Methods Study (GRAMMS) guidelines [23] to ensure transparency and rigor in describing the design, integration, and interpretation of both components.

### 2.1. Quantitative Component

The quantitative phase of the study involved administering a survey to all eligible quality nurses working in public hospitals in Kuwait. The inclusion criteria were full-time quality nurses with at least one year of experience in a healthcare setting. We excluded part-time and seconded nurses. A total population sampling approach was used. At the time the data were collected, 85 nurses were assigned to quality departments across public hospitals in Kuwait. Of these, 20 nurses were excluded because they were either employed part-time or were seconded staff and therefore did not meet the eligibility criteria. Consequently, 65 eligible full-time quality nurses were invited to participate; all 65 completed the survey, yielding a response rate of 100%. Because this study surveyed the entire eligible population of full-time quality nurses working in public hospitals in Kuwait, no separate sample-size calculation was performed.

Data were collected using a three-part questionnaire (Supplementary File S1):

1. Demographic data, including age, sex, marital status, years of experience, and level of education.
2. The six-item Turnover Intention Scale (TIS-6) [24] is a shortened version of the 15-item TIS developed by Roodt (2004) [25]. The TIS-6 assesses employees’ intentions to stay with or leave an organization and was selected based on its established reliability (Cronbach’s α = 0.80) and validity [24]. Not all participants were Arabic speakers, so the original English version was administered because it was felt that all recipients would be sufficiently proficient in English to complete the questionnaire.
3. Reasons for intending to leave. The items were generated from a comprehensive review of the literature [1,26,27,28] and were reviewed by five quality consultants from the Quality and Accreditation Directorate to assess their clarity, relevance, and comprehensiveness. Feedback from the reviewers was incorporated into the final questionnaire. No formal content validity index (CVI) assessment or pilot testing was undertaken.

The survey was created using Google Forms. Participation was entirely voluntary. Nurses were informed that choosing not to participate or withdrawing before submitting the survey would have no effect on their employment, professional standing, or performance appraisal. Informed consent was obtained after explaining the purpose and potential benefits of the study. The Nursing Director at the Quality and Accreditation Directorate distributed a link to the survey via WhatsApp. All quality nurses working in public hospitals were functionally affiliated with the Quality and Accreditation Directorate and belonged to a single professional WhatsApp group. Consequently, the survey invitation reached the entire eligible population simultaneously. According to the electronic timestamps, survey responses were submitted on 12–13 November 2022. Only the aggregate number of completed responses was available to the Nursing Director; individual respondents’ identities were not visible. No reminder messages were sent, and only a thank-you message was communicated after participation.

The TIS-6 uses a semantic differential scale as follows:

- Items 1, 3, and 4: 1 = never to 5 = always
- Item 2: 1 = very satisfying to 5 = totally dissatisfying
- Item 5: 1 = highly unlikely to 5 = highly likely
- Item 6: 1 = always to 5 = never

Item scores were summed to calculate a total score ranging from 6 to 30, with higher scores indicating stronger turnover intention. The scale midpoint is 18 (3 × 6). According to the scoring guidance provided by the original developer of the TIS-6, as reported in reference [29], scores below 18 indicate a desire to stay, whereas scores above 18 indicate a desire to leave the organization. The >18 threshold was therefore used in this study to describe the proportion of participants whose scores were consistent with an intention to leave. The continuous TIS-6 score was retained for descriptive statistics and all between-group analyses.

Data from completed surveys were cleaned in Microsoft Excel, coded, and analyzed using SPSS version 23 (significance level α = 0.05). The reliability of the TIS-6 was assessed using Cronbach’s alpha. Data normality was tested using the Kolmogorov–Smirnov and Shapiro–Wilk tests. Descriptive analyses were performed using frequencies and percentages for categorical variables. Continuous variables with approximately normal distributions were summarized using means and standard deviations, whereas non-normally distributed variables were summarized using medians and interquartile ranges. Bivariate non-parametric tests (Mann–Whitney U and Kruskal–Wallis H tests) were used to examine differences in turnover-intention scores across sociodemographic groups. No multivariable prediction model was fitted because of the relatively small size of this specialist workforce. Therefore, age and work experience were examined separately in the bivariate analyses, and multicollinearity assessment was not applicable.

### 2.2. Qualitative Component

The qualitative phase involved in-depth, semi-structured interviews with nurses recruited from the quantitative sample. Because the survey was anonymous, individual TIS-6 scores could not be linked to participants’ identities. After completion of the survey, all respondents were invited to indicate voluntarily whether they had an intention to leave and were willing to participate in an individual interview to explore the reasons underlying their intention. Seven nurses indicated that they had an intention to leave and agreed to participate. These participants were purposively selected because they could provide rich, relevant information regarding the phenomenon of interest and also represented variation in years of experience and educational background (from diploma to postgraduate degrees) [30].

Given the specialized and relatively homogeneous nature of the study population, the qualitative phase prioritized depth of experiential understanding over broad representativeness. Interview data were reviewed during data collection to identify recurring and newly emerging issues. Recurrence of the principal issues was evident by the sixth interview, and the seventh interview did not generate additional codes or subthemes. Interviewing concluded after the available purposive sample had been completed. This pattern was consistent with thematic saturation within this focused sample, although no formal prospective saturation assessment or saturation log was used.

The qualitative phase used inductive thematic analysis informed by phenomenological principles [31]. The phenomenological orientation reflected the study’s focus on understanding participants’ lived workplace experiences and perceptions rather than adherence to a specific phenomenological school or formal phenomenological analytic method. It informed the use of open-ended interviewing and flexible probing to allow participants to describe and elaborate on their experiences in their own words, while the interview data were analysed inductively using thematic analysis. In-person semi-structured interviews lasted approximately 30–45 min and were conducted at a time and place convenient for the participant. All interviews were audio-recorded with consent.

Participation was voluntary. Before each interview, written informed consent was obtained, and participants were informed of their right to withdraw or decline to answer any question at any time without penalty. Confidentiality and anonymity were assured, and all identifiable data, audio recordings, and transcripts were stored securely in password-protected files accessible only to the research team. Personal data were used solely for the purposes of this study, and audio recordings and transcripts will be securely destroyed after the required retention period [32].

The interview guide consisted of two sections:

1. Demographic information (age, sex, education level, and years of experience).
2. Open-ended questions exploring participants’ experiences as quality nurses and their intentions to leave or stay.

The guide was developed from the relevant literature and the quantitative findings regarding turnover intention and reported reasons for wishing to leave, consistent with the sequential explanatory mixed-methods design. It was not possible to adapt an existing validated interview instrument because no suitable guide specific to quality nurses could be identified. The developed guide was reviewed by the aforementioned five quality consultants to assess its clarity and relevance. No substantive changes were recommended, and no pilot interview was conducted. Sample questions included:

- How did you feel about the decision to leave your work as a quality nurse, and what factors influenced this decision?
- What aspects of your current position do you find most challenging or unsatisfying?
- What changes in your current position might make you reconsider leaving?
- How does work–life balance influence your decision to stay or leave?
- How do you perceive the relationship between quality nurses and management, and how does this affect your decision to remain or leave?

To ensure trustworthiness and rigor, several strategies were employed:

- Purposive sampling ensured participants could provide rich and relevant data.
- Interview questions were designed to be clear and easily understood.
- Interviews were audio-recorded and transcribed verbatim. Arabic quotations selected for publication were translated into English, then back-translated to verify the accuracy of translation.
- Researchers maintained reflexive journals throughout the process of data collection and analysis to document their assumptions, reflections, and potential sources of bias. These reflections were discussed during regular research team meetings to challenge interpretations, review emerging themes, and support balanced theme development.
- Three researchers with expertise in healthcare quality, none of whom had a direct supervisory relationship with the interview participants, independently read and manually coded all seven English-language interview transcripts. No predefined codebook or coding framework was used. Meaningful segments of the transcripts were assigned initial codes inductively, and the codebook developed iteratively as analysis progressed. Related and overlapping codes were compared, discussed, and merged where appropriate, then organized into subthemes and three broader themes. Differences in coding or interpretation were discussed among the three researchers until consensus was reached; no formal inter-coder agreement statistic was calculated. A summary of the preliminary themes was shared with participants for member checking to confirm that the interpretations accurately reflected their experiences.

## 3. Results

### 3.1. Survey Response

Of the 85 nurses working in the quality departments of Kuwait’s public hospitals at the time of data collection, 20 were excluded because they were part-time or seconded staff. All 65 eligible nurses completed the survey, yielding a response rate of 100%.

### 3.2. Sociodemographic Characteristics of Study Participants

Table 1 presents the sociodemographic characteristics of participants. The majority of respondents were female (86.2%), representing approximately 6.5 times the number of male participants. Similarly, most participants held a bachelor’s degree (87.7%), while the remainder were evenly distributed between those holding a graduate diploma or a postgraduate degree (6.2%). More than four-fifths of the sample (81.5%) were aged 30–39 years, and over half (52.3%) reported having 11–20 years of work experience. Nearly all participants were married (96.9%), with only two unmarried respondents.

### 3.3. Nurse Turnover-Intention Score (TIS-6)

The TIS-6 demonstrated good internal reliability in this study (Cronbach’s α = 0.75). The mean turnover-intention score was 21.7 (SD = 3.9), with a median of 22.0 (IQR = 19.0–24.0) and an observed range of 9–30. The mean score exceeded the scale midpoint of 18 specified in the original developer’s scoring guidance [29]. Overall, 54 of the 65 participants (83.1%; 95% CI: 72.2–90.3%) scored above 18, consistent with the developer’s interpretation of a desire to leave the organization [29]. Five participants (7.7%) scored exactly at the midpoint, whereas six (9.2%) scored below 18.

As shown in Table 1, the highest median score was observed among male participants (24.0; IQR = 18.0–24.5), followed by participants with postgraduate degrees (23.5; IQR = 18.3–28.0). However, none of the differences in median scores across sociodemographic groups were statistically significant.

Regarding individual items (Table 2), the highest mean score corresponded to Item 4: “How often do you dream about getting another job that will better suit your personal needs?” (mean = 4.0 ± 1.1). The majority of participants responded with either “4” or “5” to the following four items:

- “How often have you considered leaving your job?” (58.5%)
- “How often are you frustrated when not given the opportunity at work to achieve your personal work-related goals?” (56.9%)
- “How often do you dream about getting another job that will better suit your personal needs?” (72.3%)
- “How often do you look forward to another day at work?” (52.3%)

**Table 2:** Responses to TIS-6 items (total *n* = 65).

	Mean	(SD)	Frequency of Responses ‘4’ and ‘5’	
			*n*	(%)
TIS-6				
How often have you considered leaving your job?	3.7	(1.1)	38	(58.5)
2. How satisfying is your job in fulfilling your personal needs?	3.3	(1.0)	30	(46.2)
3. How often are you frustrated when not given the opportunity at work to achieve your personal work-related goals?	3.7	(0.9)	37	(56.9)
4. How often do you dream about getting another job that will better suit your personal needs?	4.0	(1.1)	47	(72.3)
5. How likely are you to accept another job at the same compensation level should it be offered to you?	3.5	(1.1)	30	(46.2)
6. How often do you look forward to another day at work?	3.6	(0.7)	34	(52.3)

SD: standard deviation; *n*: number; %: percentage.

### 3.4. Reasons for Leaving

The frequencies of participants’ reasons for wishing to leave are summarized in Table 3. The top six reasons were all work-related. The most frequently cited factor was salary (60%), followed by an uncomfortable or stressful work environment with no teamwork (49.2%) and lack of recognition and reward (44.6%). The most commonly reported personal reason was “Accepting an offer from another organization outside Kuwait” (15.4%).

### 3.5. Qualitative Analysis (Inductive Thematic Analysis Informed by Phenomenological Principles)

The qualitative phase was undertaken to further explain the quantitative findings by exploring participants’ experiences and perceptions of the workplace factors underlying their turnover intention. Analysis of the interview data generated three overarching themes and 12 subthemes, which provided contextual understanding of the reasons reported in the questionnaire and highlighted how these factors were experienced in daily practice. It is worth noting that the analysis followed a nonlinear, iterative process, involving continuous movement between the whole dataset and individual parts. Through reflection and discussion, the research team reached agreement on the final themes and subthemes. The demographic characteristics of interview participants are shown in Table 4.

Thematic analysis identified three overarching themes describing participants’ experiences related to their intentions to leave their employment: work environment factors, compensation and benefits, and personal reasons. Representative quotations illustrating each subtheme are presented in Supplementary File S2, with two quotations provided for each subtheme. Selected quotations are presented below to illustrate the main findings.

#### 3.5.1. Theme 1: Organizational and Workplace Factors

##### Lack of Support

Participants reported a lack of organizational support and a workplace culture that undervalued their contributions. They described feeling unappreciated and blamed by management, which fostered a sense of helplessness. Such a negative culture was perceived as a major factor driving the desire to leave.

*“Our quality physician delegates tasks for us that are not related to quality, and we cannot refuse, as there is no support from management… In spite of all the hard work we are putting into our work, we are still blamed for everything.”*

(Participant 3)

##### Unprofessional Behavior

Participants described experiencing unprofessional behavior, including discrimination, verbal abuse, harassment, and public criticism. Several nurses recounted instances where doctors shouted at or shamed them publicly. These experiences made those nurses feel marginalized and excluded, reinforcing their perception that they did not belong to the organization. They attributed this to inequitable management practices and perceived stigma associated with their nationality or professional role as nurses.

##### Poor Communication

Participants highlighted poor communication as a major source of frustration, citing unclear instructions, inadequate feedback, and a lack of transparency. These communication barriers contributed to dissatisfaction and a desire to seek employment in environments they perceived to be more communicative.

##### Lack of Self-Actualization

Participants felt their contributions were not adequately recognized or rewarded. They also reported limited access to training and professional development opportunities, leaving them feeling ill-equipped for their roles and unable to achieve personal or professional growth.

##### Role Ambiguity

Many participants described a lack of clarity regarding their job responsibilities and role expectations. The absence of a clear job description and the assignment of unrelated administrative duties led to confusion and frustration about their core functions.

*“We are handling several tasks not related to our job, and we do not have a clear, valid job description.”*

(Participant 2)

##### Workload

Participants consistently mentioned excessive workloads compounded by staff shortages. They reported long working hours and difficulty in completing their duties within regular shifts, contributing to burnout and job dissatisfaction.

##### Emotional Exhaustion

Participants expressed emotional fatigue stemming from a demanding work environment and insufficient managerial support. They described feelings of hopelessness, frustration, and disengagement, often using expressions such as feeling “empty” or “drained.” Many expressed a desire for a calmer and more supportive organizational culture.

*“Quality nurses are human beings. We are frustrated due to a lot of reasons: no appreciation or reward, no support.”*

(Participant 7)

##### Duty Hours

Some participants indicated that predictable duty hours were a positive factor encouraging them to remain in their current positions. Quality nurses generally worked regular daytime office hours (morning or straight duty), unlike many clinical nurses who worked rotating shifts, evenings, nights, weekends, and public holidays. Participants regarded this predictable schedule as one of the main advantages of the role. Participants specifically described regular morning or straight duty as a reason to remain in the role.

*“Morning duty is the reason to stay till now.”*

(Participant 5)

#### 3.5.2. Theme 2: Compensation and Benefits

##### Lack of Medical Insurance

Participants voiced dissatisfaction with the absence of employee healthcare benefits, particularly medical insurance. They noted that this lack of coverage placed a financial burden on them and their families.

##### Low Salary

Participants highlighted perceived inequities in pay and benefits compared with nurses working in clinical roles. They expressed frustration over lower salary scales, reduced allowances, and the absence of financial recognition for their responsibilities.

*“We suffer from inequality in salary… they cut all the other allowances, and I do not know why.”*

(Participant 7)

#### 3.5.3. Theme 3: Personal Reasons

##### Accepting an Offer from Another Organization

Several participants indicated that they had been approached by other healthcare organizations offering higher salaries, better benefits, or more promising opportunities for career advancement. If the new offers aligned more closely with their professional aspirations, they considered leaving their current roles.

##### Personal Responsibility

Personal obligations, such as financial commitments and family responsibilities, were also cited as influencing participants’ decisions to stay or leave their organizations.

### 3.6. Integration of Quantitative and Qualitative Findings

The qualitative findings complemented and expanded on the quantitative results by providing contextual insight into how participants experienced the workplace factors reported in the survey. Whereas the quantitative phase identified dissatisfaction with salary, poor work environment, and lack of recognition as common reasons for intending to leave, the qualitative phase illustrated how these factors manifested in daily practice through a culture of blame, verbal mistreatment, inadequate communication, unclear role expectations, emotional fatigue, and perceived inequity. In addition, favorable duty hours emerged from the qualitative findings as a potential factor supporting participants’ willingness to remain in their current roles, a dimension that was less apparent in the quantitative phase.

Integration of the two phases indicated that the high turnover intention observed quantitatively coexisted with multiple organizational and interpersonal challenges reported during the interviews. Within the JD–R framework, participants described concurrent job demands—including workload, role ambiguity, verbal mistreatment, emotional exhaustion, and financial dissatisfaction—alongside insufficient organizational resources such as managerial support, professional recognition, effective communication, and career development opportunities. The qualitative findings thus contextualized the quantitative results by illustrating workplace experiences underlying the commonly reported reasons for intending to leave and identifying dimensions not fully captured by the survey. In particular, the interviews revealed that dissatisfaction with salary extended beyond pay level to include perceived inequity and loss of financial benefits, while favorable morning duty hours emerged as a practical consideration supporting participants’ willingness to remain in their roles.

## 4. Discussion

Healthcare organizations increasingly depend on a skilled workforce to sustain patient safety, quality improvement, regulatory compliance, and continuous service improvement [33,34]. Consequently, attracting and retaining qualified healthcare professionals has become a strategic priority worldwide amid persistent workforce shortages [35,36,37]. Moreover, effective staffing requires accurate forecasting and proactive management of workforce turnover [37].

The present study revealed a high level of turnover intention among quality nurses working in public hospitals. This finding is consistent with previous studies [9,38,39,40]; however, it is important to note that those studies primarily focused on nurses engaged in clinical care. This highlights a gap in the literature, and there is an evident lack of research exploring turnover intentions among quality specialists in general, and particularly among those with a background in nursing.

Turnover intention is widely regarded as one of the strongest predictors of actual employee turnover [10]. It reflects behavioral, cognitive, and psychological processes through which dissatisfaction with work or organizational conditions can evolve into intentions to seek alternative employment and, ultimately, actual turnover in staff for an organization [24,25].

Previous studies have shown that demographic characteristics such as sex [1,20,41,42], marital status [1,20,43] and level of education [1,20,41] are all associated with intentions of individuals to leave an organization. Moreover, some studies of bedside nurses [40,42,44] have found that turnover intention can be predicted based on demographic variables. Interestingly, contrary to these earlier findings, the current study did not identify any statistically significant differences in turnover-intention scores across the sociodemographic groups examined. The absence of significant differences might reflect the small and relatively homogeneous census population, as well as the similar organizational context of a specialist workforce; the subgroup analyses also had limited statistical power.

Responses to the individual TIS-6 items indicated that many participants wished to work in positions that better met their personal and professional needs. However, the quantitative nature of the instrument did not permit exploration of what those specific needs entailed. Consequently, the focus of the qualitative phase shifted toward exploring factors reported in the survey as reasons for turnover intention, rather than undisclosed factors that might encourage employees to remain.

Poor working conditions, limited career development opportunities, and inadequate remuneration have consistently been associated with turnover intention among healthcare professionals [45]. The present study similarly found that organizational and work-related factors were reported more frequently than personal reasons for turnover intention, suggesting that organizational-wide interventions might offer greater opportunities for improving retention than strategies focused solely on personal factors. Nevertheless, organizational change is inherently complex, often requiring sustained leadership commitment and system-level improvements [46].

Integrating the quantitative and qualitative findings demonstrated that the interviews not only confirmed the survey results but explained how the reported workplace factors were experienced in everyday practice. For example, the qualitative findings provided important contextual insight into how these organizational factors were experienced by quality nurses. Participants described inadequate organizational support, ineffective communication, role ambiguity, heavy workloads, emotional fatigue, and perceived inequitable treatment as interconnected aspects of their working environment rather than isolated issues. Reports of exclusion, public criticism, and unequal treatment also suggest that perceptions of organizational justice and psychological safety might have been relevant to turnover intention, reflecting the extent to which nurses felt respected, supported, and valued within the organization.

Viewed through the JD–R model [22], workload, role ambiguity, and emotional fatigue are demands of the job, whereas managerial support, professional recognition, career development opportunities, effective communication, and predictable duty hours represent job resources that might help buffer occupational strain [47,48,49]. This framework provides a coherent interpretation of the convergence between the quantitative and qualitative findings. Rather than operating independently, the organizational factors identified in this study appear to reflect an overall imbalance between job demands and available resources within the role of the quality nurse. Although the cross-sectional design precludes causal inference, the findings suggest that organizational conditions perceived by participants are perhaps more relevant to turnover intention than demographic characteristics in this specialist workforce.

Supporting our findings, the literature consistently identifies organizational factors as important influences on nurses’ turnover intention. The work environment has been widely recognized as a major determinant [50,51], with lack of organizational support also emerging as a key contributor [52]. Supportive work environments help reduce stress, burnout, and dissatisfaction [53,54], whereas frustration within the workplace has been associated with increased turnover intention [55]. Similarly, unprofessional behavior—including bullying, harassment, discrimination, and incivility—has been shown to adversely affect nurses’ well-being, mental health, job performance, and organizational commitment, thereby increasing turnover intention [56,57,58,59]. Poor communication between nurses, colleagues, and supervisors represents another important organizational factor. Ineffective communication can contribute to misunderstandings and conflict in the workplace, reducing job satisfaction and increasing turnover intention, whereas effective communication has been associated with improved job satisfaction and greater staff retention [57,60]. In addition, limited career development opportunities and role ambiguity have been consistently associated with nurses’ turnover intention [61,62,63,64]. Similar challenges relating to role ambiguity, organizational support, and workplace conditions have also been reported among nurses in other Gulf settings [20].

Role ambiguity might manifest differently for quality nurses than for bedside nurses. In the literature, bedside nurses reported three defining attributes of role ambiguity: information deficiency, lack of clarity, and unpredictability [65]. Despite this, bedside nursing roles are usually linked to defined clinical tasks and unit-based supervision when compared to roles of quality nurses. In the current study work context, quality nurses have transitioned from traditional bedside care to quality management roles. They work across departments, coordinating quality improvement, accreditation, patient safety, regulatory compliance, and clinical auditing activities, and might report through more than one administrative line. Their position often requires collaboration with managers, physicians, and frontline clinical teams, albeit with limited formal authority over many of the processes they are expected to improve. Consequently, quality nurses can experience competing expectations, unclear role boundaries, and professional frustration if quality initiatives are not fully understood or supported by frontline staff. As reported in an integrative review, transitioning from traditional bedside care to a non-clinical role introduces organizational stressors that differ from those encountered in routine clinical practice [66]. In the present study, these challenges may have been further compounded by the absence of an approved job description, which could have helped quality nurses define their scope of practice, manage scope creep, navigate conflicting directives from multiple authorities, and establish clearer lines of communication and reporting.

Work overload emerged as an important factor influencing the nurses’ intentions to leave. This supports prior findings linking high workloads to burnout and reduced job satisfaction [67,68]. Emotional fatigue also emerged as a frequently reported contributor to intention to leave, consistent with earlier studies identifying it as a strong predictor of nurse turnover [69,70]. In addition, personal reasons, such as familial responsibilities and receiving offers of employment from other organizations, contributed to greater intention to leave, which is consistent with what the literature reports [71,72]. Moreover, compensation and benefits, including low salary, inequitable pay distribution, and lack of medical insurance, were also frequently cited as contributors to nurses’ intentions to leave [73,74].

Financial dissatisfaction emerged as one of the most frequently reported contributors to turnover intention. In fact, quality nurses are excluded from the allowances allocated to nursing staff who assume supervisory responsibilities concurrently with their clinical duties and pedagogical training roles. Furthermore, they are denied occupational hazard allowances designated for workplace risks, including physical danger, infection, environmental contamination, and acoustic pollution. Additionally, those transitioning from specialized units—such as Intensive Care Units and Operating Rooms—experience a reduction in their department-specific salary premiums. Despite assuming responsibilities related to quality improvement, accreditation, regulatory compliance, and patient safety, they receive lower pay. However, the qualitative findings indicated that dissatisfaction reflected perceived inequity rather than salary level alone. These perceptions of inequitable remuneration and professional recognition could be interpreted as being more influential to turnover intention than absolute salary levels alone, through an undermining of employees’ sense of fairness and the value attributed to their role. Several Gulf Cooperation Council countries have introduced workforce initiatives to strengthen nurse recruitment, retention, leadership development, and career progression; however, organizational support, career development opportunities, and work environment continue to influence nurse retention across the region [20,75,76]. Within this context, the present findings suggest that strengthening internal pay equity and professional recognition for quality nurses could be an important component of broader workforce-retention strategies in Kuwait.

Duty hours emerged as an important consideration in participants’ accounts of their intention to leave or remain. In the current study, some participants described regular morning duty hours (07:00 a.m.–02:00 p.m.) as a reason for remaining in their jobs; indeed, previous studies have shown that irregular duty hours increase turnover intention [11,77]. Although this aspect was not explored in greater depth, the identification of favorable duty hours as a reported reason for remaining opens the door to new possibilities. Investigating and reinforcing factors that encourage nurses to remain in their roles might offer a more feasible and practical approach to reducing turnover rates than focusing solely on eliminating factors that drive them to leave. This finding also illustrates that retention is influenced not only by reducing factors that encourage employees to leave but also by strengthening conditions that make them want to remain. Future studies should therefore examine both drivers of turnover intention and facilitators of retention.

Collectively, participants reported organizational and structural conditions more prominently than isolated personal factors in relation to turnover intention. Poor communication, lack of support, role ambiguity, emotional exhaustion, and inequitable treatment appear to reflect broader deficiencies in organizational culture and leadership practices [78,79,80,81,82]. These findings reinforce the importance of system-level interventions that address workplace culture, managerial engagement, and professional recognition rather than relying solely on individual-focused retention strategies.

The findings have several practical implications. Healthcare organizations could consider strengthening organizational support, improving communication, clarifying role expectations, expanding professional development opportunities, managing workload, and reviewing remuneration structures as potential approaches to supporting retention. Given the organizational nature of many of the reported concerns, system-level interventions targeting workplace culture and professional recognition might warrant greater consideration alongside approaches focusing solely on individual employees.

### 4.1. Strengths and Limitations

This study represents the first nationwide investigation of turnover intention among nurses working in quality management roles in public hospitals in Kuwait. The cross-sectional quantitative phase efficiently assessed the prevalence of turnover intention and the participants’ reported reasons for intending to leave within the entire eligible population of full-time quality nurses working in public hospitals [83]. The use of the internationally recognized TIS-6 instrument also facilitates comparison with findings from other settings. The sequential explanatory mixed-methods design further strengthened the study by enabling the quantitative findings to be explored in greater depth through interviews with nurses who voluntarily reported an intention to leave, thereby providing contextual understanding of the reported experiences. Finally, the study offers novel insights into the organizational and personal experiences underlying turnover intention in this little-studied specialist nursing workforce.

Despite these strengths, several limitations should be acknowledged. The reliance on self-reported data introduces the possibility of social-desirability response bias [84]. The cross-sectional design and descriptive analytical approach do not permit causal inference or identification of independent statistical predictors of turnover intention. Although the quantitative phase included the entire eligible population of full-time quality nurses working in public hospitals in Kuwait, the relatively small size of this specialist workforce limits the generalizability of the findings to other healthcare systems and healthcare settings. In addition, the limited sample size reduced the statistical power available for stable multivariable modelling; consequently, the quantitative analyses were limited to descriptive and bivariate comparisons and do not identify independent predictors of turnover intention. The limited body of research specifically addressing quality nurses also restricted the availability of directly comparable studies. The TIS-6 was administered in English and was not culturally validated specifically for the Kuwaiti context. Although the developer-recommended midpoint threshold was used to describe the proportion of participants whose TIS-6 scores were consistent with an intention to leave, dichotomizing a continuous measure can obscure variation in the strength of turnover intention. The continuous TIS-6 scores were therefore retained for descriptive and between-group analyses. In addition, the researcher-developed reasons-for-leaving questionnaire underwent expert review but did not undergo formal CVI assessment or pilot testing.

The 100% response rate should be interpreted with caution. The survey link was distributed by the Nursing Director of the Quality and Accreditation Directorate, to which quality nurses were functionally affiliated. In this relatively small professional population, distribution through a senior nursing officer may have influenced perceptions regarding participation and potentially contributed to the 100% response rate, despite participation being explicitly voluntary. It may also have introduced perceived hierarchical influence or social desirability bias in participants’ responses. However, the organizational and workplace factors assessed—including managerial support, communication, unequal treatment, remuneration, and working conditions—primarily concerned participants’ experiences within the hospitals where they were operationally based rather than the Directorate itself. Individual responses were anonymous, and the Nursing Director had access only to the aggregate number of completed questionnaires. Nevertheless, an influence of the recruitment procedure on participation or responses cannot be completely excluded.

Furthermore, because the qualitative phase included only participants who reported an intention to leave, it could explore experiences underlying turnover intention but could not compare these experiences with those of nurses intending to remain or establish factors associated with retention. Objective salary data, cost-of-living measures, and actual working hours were not collected; therefore, the findings relate to perceived remuneration and self-reported duty patterns.

### 4.2. Implications for Practice and Policy

In the context of Kuwait’s public healthcare system, the following strategies should be regarded as options for consideration and evaluation rather than interventions proven by this cross-sectional study.

#### 4.2.1. Organizational and Leadership Support

Hospital administrators could consider strengthening organizational and managerial support for quality nurses. The study identified lack of support, unprofessional behavior, and poor communication as prominent organizational factors associated with turnover intention. The creation of regular communication mechanisms between quality nurses, hospital management, and clinical departments might improve collaboration and ensure that concerns are identified and addressed promptly. Leadership development programs that promote equitable management practices, constructive feedback, professional recognition, and respectful workplace behaviors could also be considered to support staff retention.

#### 4.2.2. Clarifying Roles and Enhancing Professional Development

Ambiguity in job responsibilities and limited opportunities for professional growth emerged as significant challenges. Hospital management could establish and communicate clear job descriptions (currently not issued at these hospitals) that define the responsibilities, reporting relationships, and scope of practice of quality nurses, while reducing assignment of duties unrelated to their specialist role. Structured orientation, continuing professional development, mentorship, and support for relevant quality-management and patient-safety certifications might strengthen professional competence and role identity. Developing transparent career progression pathways for quality nurses could further enhance long-term retention.

#### 4.2.3. Workload Management and Emotional Well-Being

Heavy workloads and emotional fatigue were recurrent issues reported by participants. Organizations could assess staffing levels, workload distribution, and allocation of quality-management responsibilities to ensure that expectations are aligned with the available personnel and protected time. Where appropriate, workload monitoring mechanisms could be introduced to identify sustained excessive demands. Stress-management, counselling, and peer-support programs might provide useful complementary support but should not replace action on structural workload and staffing issues.

#### 4.2.4. Compensation and Benefits

Financial dissatisfaction was among the most frequently cited reasons for intention to leave. The qualitative findings suggested that dissatisfaction stemmed largely from perceived inequity in salaries and allowances compared with their peers working in clinical nursing roles, despite undertaking responsibilities related to quality improvement, accreditation, regulatory compliance, and patient safety, rather than from salary level alone. Relevant health and civil-service authorities could therefore review salary scales, allowances, and employee benefits to assess whether they are equitable and appropriately aligned with the responsibilities and scope of the quality-nurse role. Because this study did not collect objective salary or cost-of-living data, any regulatory reform should be informed by a separate remuneration review.

#### 4.2.5. Promoting Flexible Work Arrangements

Predictable duty hours emerged from participants’ accounts as a factor that might support willingness to remain in their current roles. Maintaining regular daytime schedules for quality nurses, where operationally feasible, might help preserve work–life balance while supporting the continuity of quality-management activities. The study did not evaluate remote working arrangements or alternative scheduling models; therefore, any broader workforce-flexibility initiatives should be evaluated separately before implementation.

#### 4.2.6. Quality of Life as a Research Priority

Quality of life was not measured in this study; therefore, its relationship with turnover intention should be examined in future research rather than assumed in current workforce policy. Future studies could also evaluate interventions aimed at improving organizational support, role clarity, workload management, and professional development to determine their effectiveness in retaining quality nurses.

### 4.3. Future Research

For future research, longitudinal studies are recommended to examine changes in turnover intention and quality of life among quality nurses over time, thereby capturing the evolving nature of these interrelated constructs. Investigating the relationship between quality of life and intention to leave would provide valuable insights into the relationship between nurses’ overall well-being and their retention decisions. Moreover, using mixed-methods designs with larger and more diverse samples would allow for a deeper exploration of the contextual and experiential factors underlying these intentions. Replicating this study across different healthcare sectors and regions in Kuwait—and potentially in comparable healthcare systems—would further enhance the generality of the findings and inform the development of evidence-based strategies to improve both nurse well-being and retention in quality-management roles.

## 5. Conclusions

This nationwide sequential explanatory mixed-methods study provides the first evidence on turnover intention among quality nurses in Kuwait’s public hospitals. Turnover intention was highly prevalent, and participants consistently reported organizational and work-related concerns, including poor workplace culture, inadequate support, role ambiguity, workload pressures, emotional exhaustion, and perceived financial inequity. No statistically significant differences were observed across the sociodemographic groups examined, suggesting that organizational conditions might be more salient than demographic characteristics in this specialist workforce.

Carefully evaluated improvements in role clarity, organizational support, workload management, and remuneration might help support the stability of quality management, accreditation, and patient safety activities in Kuwait’s public hospitals. Future studies should use longitudinal and multicenter designs and examine quality of life and actual turnover outcomes.

## Figures and Tables

**Table 1:** Sociodemographic characteristics, TIS-6 scores, and prevalence of intention to leave among participants.

	*n*	%	95% CI	Median	[IQR]	*p*
All	65	100.0		21.7 ^†^	(3.9) ^‡^	
				22.0	[19.0–24.0]	
Sex						0.724
Female	56	86.2		22.0	[19.0–24.0]	
Male	9	13.8		24.0	[18.0–24.5]	
Age						0.711
20–29 years	3	4.6		22.0	[15.0–]	
30–39 years	53	81.5		22.0	[19.0–24.0]	
40–49 years	7	10.8		20.0	[19.0–24.0]	
Over 50 years	2	3.1		20.5	[20.0–]	
Marital status						0.675 *
Married	63	96.9		23.0	[20.0–]	
Unmarried	2	3.1		22.0	[19.0–24.0]	
Highest educational credential						0.768
Graduate Diploma	4	6.2		22.0	[21.3–25.0]	
Bachelor	57	87.7		22.0	[19.0–24.0]	
Post-graduate	4	6.2		23.5	[18.3–28.0]	
Work experience						0.289
1–10 years	28	43.1		21.0	[18.0–24.0]	
11–20 years	34	52.3		23.0	[19.8–24.0]	
21 years or more	3	4.6		20.0	[20.0–]	
TIS-6 score relative to scale midpoint **						
>18	54	83.1	[72.2%, 90.3%] ***			
=18	5	7.7				
<18	6	9.2				

*n*: number; %: percentage; CI: confidence interval; IQR: interquartile range; *p*: *p*-value; ^†^: mean; ^‡^: standard deviation; *: Exact significance is displayed; **: According to the original developer’s scoring guidance [29], scores > 18 indicate a desire to leave and scores < 18 indicate a desire to stay; a score of exactly 18 represents the scale midpoint and was not assigned to either category. ***: 95% CI was calculated using the Wilson Score method. The overall TIS-6 score is presented as mean ± standard deviation (SD), whereas subgroup TIS-6 scores are presented as median [interquartile range] because non-parametric group comparisons (Mann–Whitney U and Kruskal–Wallis H tests) were performed.

**Table 3:** Reasons for nurses’ intention to leave (total *n* = 65).

	*n*	(%)
Personal reasons		
Accepting offer from other organizations outside Kuwait	10	(15.4)
Follow spouse (husband or wife) or family, get married, do a pregnancy program	3	(4.6)
Accepting offer from other organizations inside Kuwait	1	(1.5)
Continue their study	0	(0.0)
Co-worker’s influence	0	(0.0)
Work-related reasons		
Salary	39	(60.0)
Uncomfortable working environment/stressful/no teamwork	32	(49.2)
Less recognition and reward	29	(44.6)
Feeling insecure, Lack of support	16	(24.6)
No respect, No appreciation	12	(18.5)
No investment in nurses, No career development (promotions)	12	(18.5)
Communication gap between hospital management and quality nurses, Multiple lines of authority	9	(13.8)
No special benefits (Education support, medical insurance)	8	(12.3)
Racism, inequality	6	(9.2)
Work overload/unrelated work	5	(7.7)
No work–life balance	4	(6.2)
Blame culture/Punishment	4	(6.2)
Quality is not a priority/quality is not everyone’s responsibility	3	(4.6)
No job description/no authority	2	(3.1)

*n*: number; %: percentage.

**Table 4:** Demographic characteristics of the qualitative study participants (total *n* = 7).

	Age (Years)	Sex	Education Level	Experience (Years)
Participant 1	35	Female	Bachelor	8
Participant 2	43	Male	Bachelor	10
Participant 3	29	Female	Diploma	3
Participant 4	39	Female	Bachelor	8
Participant 5	38	Female	Masters	8
Participant 6	48	Male	Bachelor	8
Participant 7	35	Female	Bachelor	10

## Data Availability

The data supporting the findings of this study are available from the corresponding author upon reasonable request. The data are not publicly available because of privacy and ethical restrictions.

## Supplementary Materials

The following supporting information can be downloaded at https://www.mdpi.com/article/10.3390/healthcare14172669/s1. Supplementary File S1: An online form composed of three sections. The first section records the participants’ demographic information. The second section includes the Turnover Intention Scale (TIS-6). The third section includes items exploring reported reasons for intending to leave. Supplementary File S2: Summary of qualitative themes, subthemes, and representative participant quotations.

## Abbreviations

The following abbreviations are used in this manuscript:

CI	confidence interval
CVI	content validity index
IQR	interquartile range
JD–R	Job Demands–Resources
*n*	number
*p*	*p*-value
SD	standard deviation
TIS	Turnover Intention Scale
