# Supplementary material for: Intention to Leave Among Quality Nurses in Public Hospitals in Kuwait: A Sequential Explanatory Mixed-Methods Study
Source: Healthcare (Basel). 2026 Aug 22;14(17):2669. doi: 10.3390/healthcare14172669 (PMC13564753; doi:10.3390/healthcare14172669)
Supplement: Supplementary file 1 [file healthcare-14-02669-s001.zip › Supplementary File S1.pdf]

Intention to leave among quality nurses in public hospitals in Kuwait

Participation Information and Consent Statement

You are invited to participate in a research study assessing intention to leave among quality nurses working in public hospitals in Kuwait. The purpose of this study is to improve understanding of the factors influencing nurses' intention to leave their current roles.
Participation in this study involves completing an online questionnaire, which will take approximately 15 minutes to complete. Your participation is entirely voluntary. You may choose not to participate or to withdraw from the study at any time before submitting your responses, without any penalty or consequences.

No personally identifiable information will be collected, and all responses will be treated confidentially and used solely for research purposes. Data will be stored securely and accessed only by the research team.

There are no anticipated risks beyond those encountered in everyday life. Although there may be no direct benefit to you, the findings may contribute to improving workforce retention strategies and working conditions for quality nurses.

If you have any questions about the study, please contact: Hossam Elamir [dr_hossam_elamir@hotmail.com, 67767083].

By proceeding to and completing the questionnaire, you indicate that:

- You have read and understood the information provided above.
- You voluntarily agree to participate in this study.
- You are at least 18 years of age.

* Indicates required question

Part 1: Personal information

1. Age (in years) *

____

2. Sex *

Mark only one oval.

- ◯ Male
- ◯ Female

3. Marital status *

Mark only one oval.

- ( ) Married
- ( ) Unmarried

4. Work experience (in years) *

____

5. Highest educational level *

Mark only one oval.

- ( ) Graduate Diploma
- ( ) Bachelor
- ( ) Post-graduate

Part 2: Turnover Intention Scale (TIS-6)

The following section aims to ascertain the extent to which you intend to stay at the organisation. Please read each question and indicate your response using the scale provided for each question.

6. 1. How often have you considered leaving your job? *

Mark only one oval.

	1	2	3	4	5	
Nev	( )	( )	( )	( )	( )	Always

7. 2. How satisfying is your job in fulfilling your personal needs? *

Mark only one oval.

	1	2	3	4	5	
Very	( )	( )	( )	( )	( )	Totally dissatisfying

8. *

3. How often are you frustrated when not given the opportunity at work to achieve your personal work-related goals?

Mark only one oval.

	1	2	3	4	5	
Nev	◯	◯	◯	◯	◯	Always

9. *

4. How often do you dream about getting another job that will better suit your personal needs?

Mark only one oval.

	1	2	3	4	5	
Nev	◯	◯	◯	◯	◯	Always

10. *

5. How likely are you to accept another job at the same compensation level should it be offered to you?

Mark only one oval.

	1	2	3	4	5	
High	◯	◯	◯	◯	◯	Highly likely

11. *

6. How often do you look forward to another day at work?

Mark only one oval.

	1	2	3	4	5	
Alwa	◯	◯	◯	◯	◯	Never

Part 3: Reasons to leave your job

From the following options, which reasons may influence you to leave your current job as a quality nurse?

12. Please select all that apply. *

Check all that apply.

- [ ] Accepting offer from other organisations outside Kuwait
- [ ] Accepting offer from other organisations inside Kuwait
- [ ] Follow spouse (husband or wife) or family
- [ ] Continue your study
- [ ] Co-worker’s influence
- [ ] Salary
- [ ] Uncomfortable working environment
- [ ] No special benefits (Education support, medical insurance)
- [ ] Less recognition
- [ ] Lack of support
- [ ] No carrier development (promotions)
- [ ] No work-life balance
- [ ] Other: ______________________

This content is neither created nor endorsed by Google.

Google Forms
