# Supplementary material for: Intention to Leave Among Quality Nurses in Public Hospitals in Kuwait: A Sequential Explanatory Mixed-Methods Study
Source: Healthcare (Basel). 2026 Aug 22;14(17):2669. doi: 10.3390/healthcare14172669 (PMC13564753; doi:10.3390/healthcare14172669)
Supplement: Supplementary file 1 [file healthcare-14-02669-s001.zip › Supplementary File S2.pdf]

Supplementary File S2. Summary of qualitative themes, subthemes, and representative participant quotations

| Themes | Sub-themes | Similarities | Examples of participant quotations |
|---|---|---|---|
| 1. Organizational and workplace factors | 1.1. Lack of support | Participant (1, 2, 3, 4, 5, 6, 7) | **Participant 1**<br>*“When I first joined the hospital, I was assigned to ICU ward then I was transferred to quality office. In the beginning, I liked the job. Then, I was shocked with the position as there was no support. They give you the order and leave you, and you have to accomplish the task whether the work belongs or does not belong to you, without training or follow up. Even the Quality Directorate does not consider us on its staff. When we asked for our rights, the Directorate said that we did not belong to it, and we should ask our rights from the Nursing Directorate.”*<br>**Participant 7**<br>*“Nurses are forced to do work not related to them. In work there is no work support from the nursing heads. This makes staff feel that no one will protect them.”* |
| | 1.2. Unprofessional behavior | Participant (1, 2, 4) | **Participant 1**<br>*“Me personally I experienced verbal abuse from one of the doctors because I asked him to complete a report about his work. He thought that I harass him.”*<br>**Participant 4**<br>*“Doctors are not trained to respect coworkers especially nurses and have full rights to shout and shame them in front of others. Without hesitation, they would blame nurses If any mistake happened. Last but the least, partiality between Arab staff and other nationalities. For instance, in the case of providing annual leave. If you don’t consider these things, it will in turn affect staff. They will try to escape from here once they got a chance.”* |
| | 1.3. Poor communication | Participant (2, 3) | **Participant 2** |

| | | | |
|---|---|---|---|
| | | | *"There is poor communication between the quality office and our directorate. They just give order, and we did our work, but no answer or feedback from them."* **Participant 3** *"We are suffering from a communication gap between hospital management and quality nurses, and we are lost in between."* |
| | 1.4. No space for self-actualization | Participant (1, 2, 3, 4, 5, 6, 7) | **Participant 5** *"No opportunity for improvement or reward. We do all the work, but appreciation goes to the quality physician. After doing everything which is required for the hospital, the management of hospital and departments talk to us as if we did nothing."* **Participant 3** *"They are not supporting nurses to be certified Quality professionals."* |
| | 1.5. Role ambiguity | Participant (1, 2, 3, 4, 5, 6) | **Participant 2** *"Any paperwork is assigned to quality nurses. We are handling several tasks not related to our job and we don't have a clear, valid job description."* **Participant 3** *"I like being a quality nurse, but people do not understand our role as there is no proper job description in hospitals. Almost all quality nurses are doing work for other departments not related to their job work because they cannot say no. They don't have a clear decree regarding they are under whom. They get evaluated by those departments who clearly doesn't know what a quality nurse job is."* |
| | 1.6. Work overload | Participant (1, 2, 3) | **Participant 2** *"We are suffering from shortage and more workload. I like to do my best but alone I cannot."* **Participant 3** |

| | | | |
|---|---|---|---|
| | | | *"We are understaffed which leads to overload. We have to answer several calls after duty hours and during holidays."* |
| | 1.7. Emotional exhaustion | Participant (1, 2, 3, 4, 7) | **Participant 4**<br>*"I have an 8-year experience in quality office. We have good teamwork, staff are cooperating and helping each other. But still, we don't have any clear valid job description, so we have the feeling of marginalized. You have to do whatever they ask."*<br>**Participant 7**<br>*"If I tell the truth, nothing will change. This is just research. Quality Nurses are human beings. We are frustrated due to a lot of reasons: no appreciation or reward, no support."* |
| | 1.8. Duty hours | Participant (3, 5, 6, 7) | **Participant 5**<br>*"Morning duty is the reason to stay till now."*<br>**Participant 7**<br>*"I would like to stay as quality nurses only for the duty hours."* |
| 2. Compensation and benefits | 2.1. No medical insurance | Participant (1, 3, 6) | **Participant 3**<br>*"We have no insurance. If we or our kids got sick, we have to pay for everything."*<br>**Participant 6**<br>*"There is lack of medical facilities for staff and families. We have no benefit for being MOH staff."* |
| | 2.2. Low salary | Participant (1, 2, 3, 4, 5, 6, 7) | **Participant 1**<br>*"There is no allowance or incentive. We stay for long time in front of screens, but we receive no screen allowance. We also have no infection allowance, like other nurses. According to the nature of our work, we have to go in ward rounds making audits and have direct contact with patient and nurses in the clinical area. If we ask for our right, no body support us. Our salary is not enough with the high cost nowadays. What we take here is spent here."* |

| | | | |
|---|---|---|---|
| | | | **Participant 7**<br>*"We suffer from inequality in salaries. When I transferred to quality office, they cut 200 KD from my salary because no night, which is ok. However, they cut all the other allowances, and I don't know why. We are meeting patient, making audit in the clinical area daily. They do not recognize that we have right for infection and danger allowance. We spend most of the time in front of screens with no screen allowance."* |
| 3. Personal reasons | 3.1. Accepting offer from another organization | Participant (3, 4, 5) | **Participant 3**<br>*"If I got the good opportunity, I would go without any hesitation. The salary there is triple of here, you have housing allowance, good education for kids and great chance for development and medical insurance for you and your family, all my friends there are happy with these advantages, and I have to try too."*<br>**Participant 5**<br>*"If I have a chance to move from here, definitely I will."* |
| | 3.2. Personal responsibility | Participant (1, 6) | **Participant 1**<br>*"If my health condition allowed me to leave the work, I would have left the work long time ago the first opportunity, but I have to be here because of the economic condition in my country. The work environment is very stressful."*<br>**Participant 6**<br>*"Even though, my personal situation forced me to stay."* |
